# Metformin Against Herpes Zoster in Colon Cancer Patients with Type 2 Diabetes: A PSM Analysis

**DOI:** 10.7150/jca.98852

**Published:** 2025-01-01

**Authors:** Ming-Chang Li, Wan-Ming Chen, Ben-Chang Shia, Szu-Yuan Wu

**Affiliations:** 1 Graduate Institute of Business Administration, College of Management, Fu Jen Catholic University, Taipei, Taiwan.; 2 Department of Colorectal Surgery, Lo-Hsu Medical Foundation, Lotung Poh-Ai Hospital, Yilan, Taiwan.; 3 Artificial Intelligence Development Center, Fu Jen Catholic University, Taipei, Taiwan.; 4 Department of Food Nutrition and Health Biotechnology, College of Medical and Health Science, Asia University, Taichung, Taiwan.; 5 Big Data Center, Lo-Hsu Medical Foundation, Lotung Poh-Ai Hospital, Yilan, Taiwan.; 6 Division of Radiation Oncology, Lo-Hsu Medical Foundation, Lotung Poh-Ai Hospital, Yilan, Taiwan.; 7 Department of Healthcare Administration, College of Medical and Health Science, Asia University, Taichung, Taiwan.; 8 Cancer Center, Lo-Hsu Medical Foundation, Lotung Poh-Ai Hospital, Yilan, Taiwan.; 9 Centers for Regional Anesthesia and Pain Medicine, Taipei Municipal Wan Fang Hospital, Taipei Medical University, Taipei, Taiwan.; 10 Department of Management, College of Management, Fo Guang University, Yilan, Taiwan.

**Keywords:** T2DM, Colon Cancer, metformin, Diabetes-associated herpes zoster infection, dose-dependent

## Abstract

**Background:** Herpes zoster is a significant complication in cancer patients, particularly those with compromised immune systems. Previous studies have established the incidence of herpes zoster in gastrointestinal cancer patients, but there is a lack of specific analysis on colorectal cancer patients and the potential preventive role of metformin. This study aims to fill this gap by evaluating metformin's protective effects against herpes zoster in colon cancer patients with type 2 diabetes mellitus (T2DM).

**Methods:** The study cohort comprised 1,510 T2DM colon adenocarcinoma patients without distant metastasis who received standard treatments from Taiwan Cancer Registry Database. Propensity score matching (PSM) was employed to balance covariates between metformin users and nonusers. Herpes zoster infection risk was assessed using Cox regression models and incidence rate calculations. The dose-dependent effects of metformin were analyzed based on cumulative defined daily doses (cDDD).

**Results:** Metformin use was associated with a significantly reduced risk of herpes zoster infection (adjusted hazard ratio [aHR].: 0.69, 95% confidence interval [CI].: 0.51 to 0.93). A dose-dependent relationship was observed, with progressively lower aHRs across cDDD quartiles (p for trend < 0.0001). After adjusting for competing mortality risks, the aHR remained significantly lower (aHR: 0.70, 95% CI: 0.51 to 0.65). Metformin users had lower incidence rates and incidence rate ratios (IRR) of herpes zoster infection compared to nonusers (IRR: 0.75, 95% CI: 0.56 to 0.97).

**Conclusions:** We are the first to demonstrate a dose-dependent protective effect of metformin against herpes zoster in colorectal cancer patients. Our findings indicate that higher doses of metformin correlate with a greater reduction in the risk of herpes zoster.

## Introduction

Colorectal cancer is a significant healthcare concern in Taiwan, with around 15,000 new cases and 6,000 fatalities annually, making it the most prevalent malignancy in the region 1. The typical age at diagnosis is approximately 66 years for both genders, and survival rates vary based on disease stage, with a five-year survival rate of around 63.0% 1, 2. As survival prospects improve, the focus shifts towards enhancing patients' quality of life 3, particularly those dealing with cancer-induced immunosuppression. This immunosuppression often renders patients susceptible to herpes virus infections, impacting their quality of life and potentially leading to enduring post-herpetic neuralgia 4. Notably, a large prospective cohort study in Australia from 2006 to 2016 found that individuals with hematologic or solid cancer had significantly higher rates of herpes zoster infection than those without cancer, emphasizing the relevance of this issue for cancer patients 4.

Colon cancer often comes with a favorable prognosis, involving extended survival assessments over a decade to gauge treatment effectiveness 5, 6. Therefore, identifying cost-effective methods to reduce herpes zoster infection becomes crucial for enhancing the quality of life in long-term colon cancer survivors and preventing postherpetic neuralgia in this high-risk population. The association between colon cancer and herpes zoster, also known as shingles, is characterized by shared risk factors and a compromised immune system, a consequence of both cancer and its treatments like chemotherapy and radiation therapy 7, 8. Previous studies have established the incidence of herpes zoster in gastrointestinal cancer patients, but there is a lack of specific analysis on colorectal cancer patients 9, 10. This immune compromise increases susceptibility to infections and the likelihood of reactivating the varicella-zoster virus (VZV) responsible for herpes zoster 11. Given that both conditions are more prevalent in older individuals, coinciding with the age group at higher risk for colon cancer diagnosis, vigilance is essential. Emotional stress from cancer and its treatments can further weaken immunity, potentially triggering latent virus reactivation 12. Additionally, diagnostic challenges may arise as herpes zoster can mimic metastatic colon cancer lesions, leading to diagnostic confusion and treatment delays 13, 14. Although the association isn't directly causal, individuals with colon cancer should remain vigilant. Herpes zoster in cancer patients can cause severe pain, complications, and treatment interruptions, affecting overall quality of life 15, 16. Hence, meticulous management and a multidisciplinary approach are imperative to mitigate these complications and enhance patient outcomes.

The exploration of metformin's potential in mitigating herpes zoster infections among diabetic patients delves into a complex interplay involving immunity, chronic illnesses, and pharmaceutical interventions 17. Following chickenpox or varicella vaccination, the VZV can enter a latent state within sensory ganglia, potentially reactivating to cause herpes zoster, often accompanied by persistent postherpetic neuralgia 18. Despite the availability of vaccines, the global incidence of varicella and herpes zoster is increasing, particularly among older adults and individuals with cancer or autoimmune diseases, including those with Type 2 diabetes mellitus (T2DM), due to compromised immune function 19, 20. Metformin, a widely recognized antidiabetic medication, not only regulates blood sugar but also confers various health benefits, such as reducing cardiovascular risk and enhancing the immune system 21-24. Laboratory studies have indicated that metformin can strengthen T-cell immunity and mitigate inflammation 25. This has led to the hypothesis that metformin might lower the risk of herpes zoster in individuals with colon cancer undergoing anticancer treatments, prompting a study to compare the incidence of zoster between metformin users and nonusers among colon cancer patients receiving such treatments. Our research aims to uncover metformin's potential protective effects against these conditions, offering valuable insights into their management, particularly in colon cancer patients with diabetes. No prior studies have investigated the potential of metformin to prevent herpes zoster in colorectal cancer patients with T2DM. Our study uniquely highlights this aspect. Previous studies have not specifically analyzed the incidence of herpes zoster in patients with colorectal cancer 9, 10. Our study is the first to do so.

## Materials and Methods

### Study population

This population-based cohort investigation harnessed the extensive repository of Taiwan's National Health Insurance Research Database (NHIRD), encompassing a wealth of data spanning disease diagnoses, medical procedures, pharmaceutical prescriptions, demographic particulars, and beneficiary profiles 26-28. Protecting patient confidentiality, the database incorporates encrypted identifiers, while its integration with the Taiwan Cancer Registry Database (TCRD) and Taiwan's Death Registry furnishes precise insights into cancer management, disease stages, vital status, and causative factors, augmenting the study's veracity and credibility 29-32. Stringent ethical oversight was observed, with the study protocols securing the imprimatur of the Institutional Review Board of Tzu-Chi Medical Foundation (IRB109-015-B).

Our investigation exclusively targeted patients with colon adenocarcinoma devoid of distant metastases who received standard treatments and were diagnosed with T2DM during the period spanning 2008 to 2018. The established treatment paradigms for stage I-III colon cancer without metastatic spread typically involve a comprehensive approach encompassing surgical intervention, occasionally supplemented with adjuvant therapy. In Taiwan, these therapeutic protocols harmonize with the well-recognized National Comprehensive Cancer Network (NCCN) guidelines 33-36. The administration of adjuvant chemotherapy is contingent upon several factors, including cancer stage (T3 or higher) and the presence of positive lymph nodes, all serving as risk indicators 36. This therapeutic strategy is customarily recommended post-surgery to eliminate residual cancer cells that might elude detection yet harbor the potential for recurrence. Two frequently employed chemotherapy regimens are FOLFOX, a combination of 5-fluorouracil, leucovorin, and oxaliplatin, and CAPEOX, which comprises capecitabine and oxaliplatin 36. Radiation therapy, conversely, does not constitute a standard treatment for colon cancer, except in cases where a notable risk of recurrence looms due to specific factors such as T4 stage infiltration into fixed structures 37.

The study's follow-up period extended until December 31, 2021, and stringent exclusion criteria were meticulously applied: (1) Age restrictions were enforced, excluding individuals both below 20 and above 80 years of age. (2) Thorough data encompassing sex and age were prerequisites for inclusion. (3) Patients afflicted with conditions such as type 1 diabetes, hepatic failure, or undergoing dialysis were systematically excluded. (4) Individuals with a previous diagnosis of herpes zoster before the index date were not factored into the analysis. (5) To eliminate prevalent cases of T2DM, patients diagnosed prior to January 1, 2008, were methodically excluded. (6) Rigorous measures were undertaken to ascertain that metformin use commenced subsequent to the diagnosis of colon cancer, with explicit confirmation that the index date for metformin users postdated the colon cancer diagnosis. Metformin use preceding the cancer diagnosis was rigorously excluded. (7) Sole inclusion encompassed patients who adhered to standard treatments for colon cancer, and (8) individuals who received the zoster vaccine during the follow-up period were meticulously omitted from the study cohort.

Metformin use was meticulously defined as consistent daily administration for the majority of days, with an average dose equivalent to or exceeding 28 cumulative defined daily doses (cDDDs). The index date was meticulously established subsequent to the initial documented use of metformin therapy (≥ 28 cDDDs), ensuring that it unequivocally surpassed the threshold of 28 cDDDs. For metformin nonusers, the index date was harmonized to align with the identical time frame following the diagnosis of colon cancer as their corresponding metformin users. The case group exclusively consisted of T2DM patients who were administered a minimum of 28 cDDDs of metformin subsequent to their colon cancer diagnosis, while the control group comprised individuals who refrained from metformin therapy throughout the entire follow-up period.

### Study covariates and propensity score matching

In our study, T2DM colon cancer patients were stratified into distinct age groups at the index date: <60, 60-65, 66-70, and ≥71 years. To mitigate potential confounding variables, we employed Propensity Score Matching (PSM) to achieve balance between metformin users and nonusers. The covariates encompassed a comprehensive set of factors, including age, sex, income level, urbanization level, years of cancer diagnosis, AJCC clinical stage (8^th^ edition), numbers of antidiabetic drugs employed (as a proxy for T2DM severity), utilization of antidiabetic medications, diabetes severity (quantified via adapted Diabetes Complications Severity Index [aDCSI]. scores), presence of coexisting comorbidities, Charlson comorbidity index (CCI) scores, and additional medications (such as statins, aspirin, immunosuppressants, and systemic corticosteroids). PSM was diligently applied to harmonize all covariates between metformin users and nonusers, given their disparate antidiabetic drug usage patterns. Medication use was consistently defined as a minimum of 28 cDDDs post-diagnosis of colon cancer. The index date was stipulated subsequent to the initiation of metformin treatment (≥28 cDDDs), and metformin users and nonusers were matched based on variables collected at this specific index date. Prior to any analytical procedures, we conducted baseline matching between metformin users and nonusers to ensure the equivalence of data at the index date. To prevent redundancy in multivariate analyses, duplicate comorbidities were meticulously excluded from the calculation of CCI scores. Comorbidities were ascertained from diagnostic codes (International Classification of Diseases, Ninth Revision, Clinical Modification, and International Classification of Diseases, Tenth Revision, Clinical Modification) documented in inpatient records or the presence of a minimum of two outpatient visits within a one-year timeframe preceding the index date. Continuous variables were aptly presented with appropriate metrics, such as mean ± standard deviation or median (first quartile and third quartile).

### Outcome variables

Our primary objective was to evaluate the risk of herpes zoster infection, closely monitoring occurrences from the index date until either herpes zoster diagnosis or the study's endpoint on December 31, 2021. The diagnosis of herpes zoster was based on clinical presentation and confirmed through medical records coded with the ICD-9-CM code 053 and ICD-10-CM code B02. The lesions were typically located on the skin following the nerve distribution pattern and were not found within the colon tissues, as the clinical manifestation of HZ within internal organs is exceedingly rare and not within the scope of this study. Furthermore, we investigated secondary outcome measures, encompassing the incidence rate (IR) and incidence rate ratio (IRR) of herpes zoster infection within our cohort.

### Metformin exposure

Metformin prescriptions in our study were precisely coded using the Anatomical Therapeutic Chemical (ATC) classification system 38, enabling the precise retrieval of pharmaceutical claims data from the NHIRD. To investigate potential dose-response associations, patients were stratified into four subgroups, defined by quartiles (Q) of cDDD. Our statistical models included thorough adjustments for the covariates mentioned above, ensuring a rigorous and comprehensive analysis of the data.

### Statistical analysis

**Figure 1:**
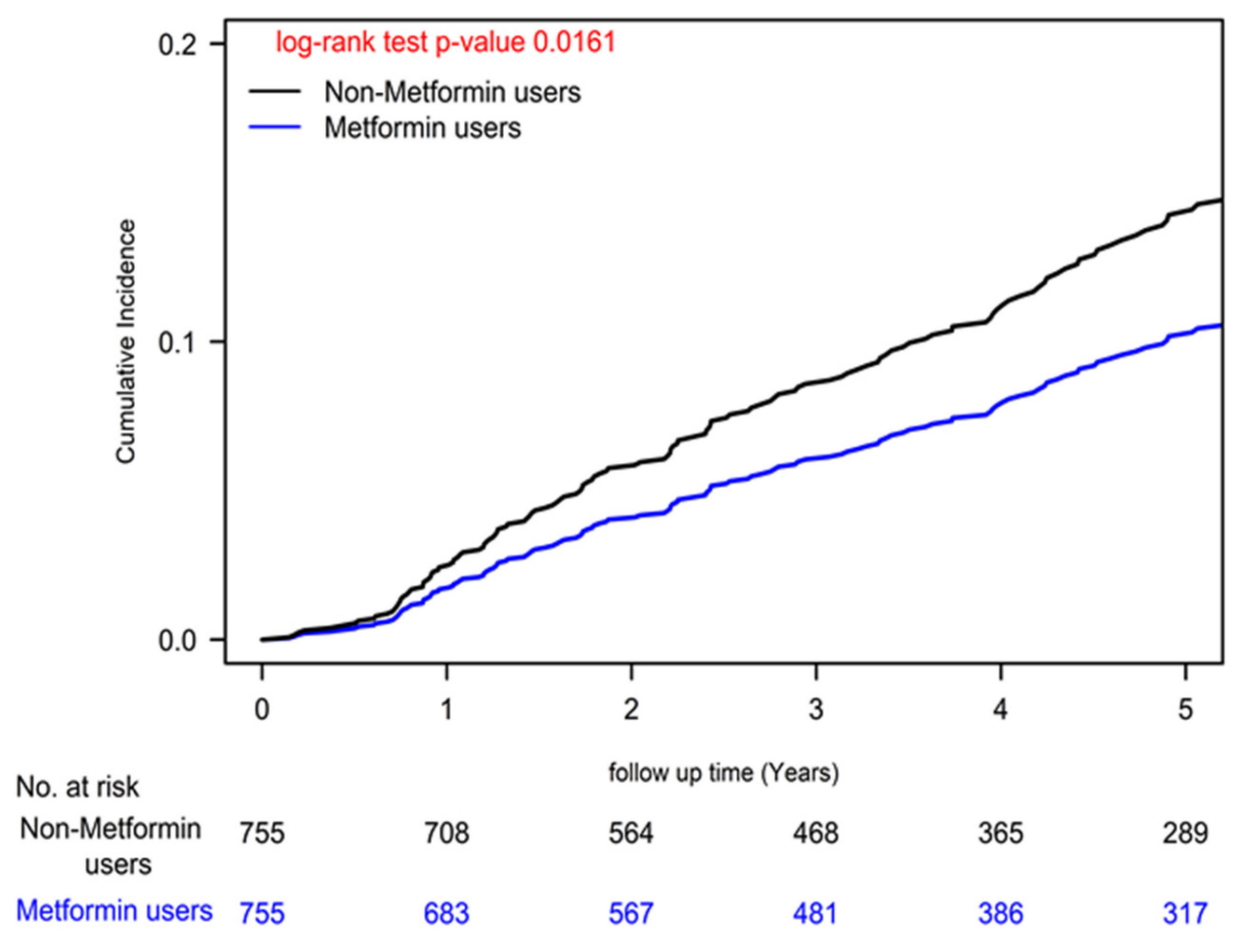
Cumulative Incidences of Herpes Zoster Infection in Patients with Type II Diabetes and Colon Cancer Undergoing Curative Standard Treatments: A Comparison Between Metformin Users and Non-Users.

**Figure 2:**
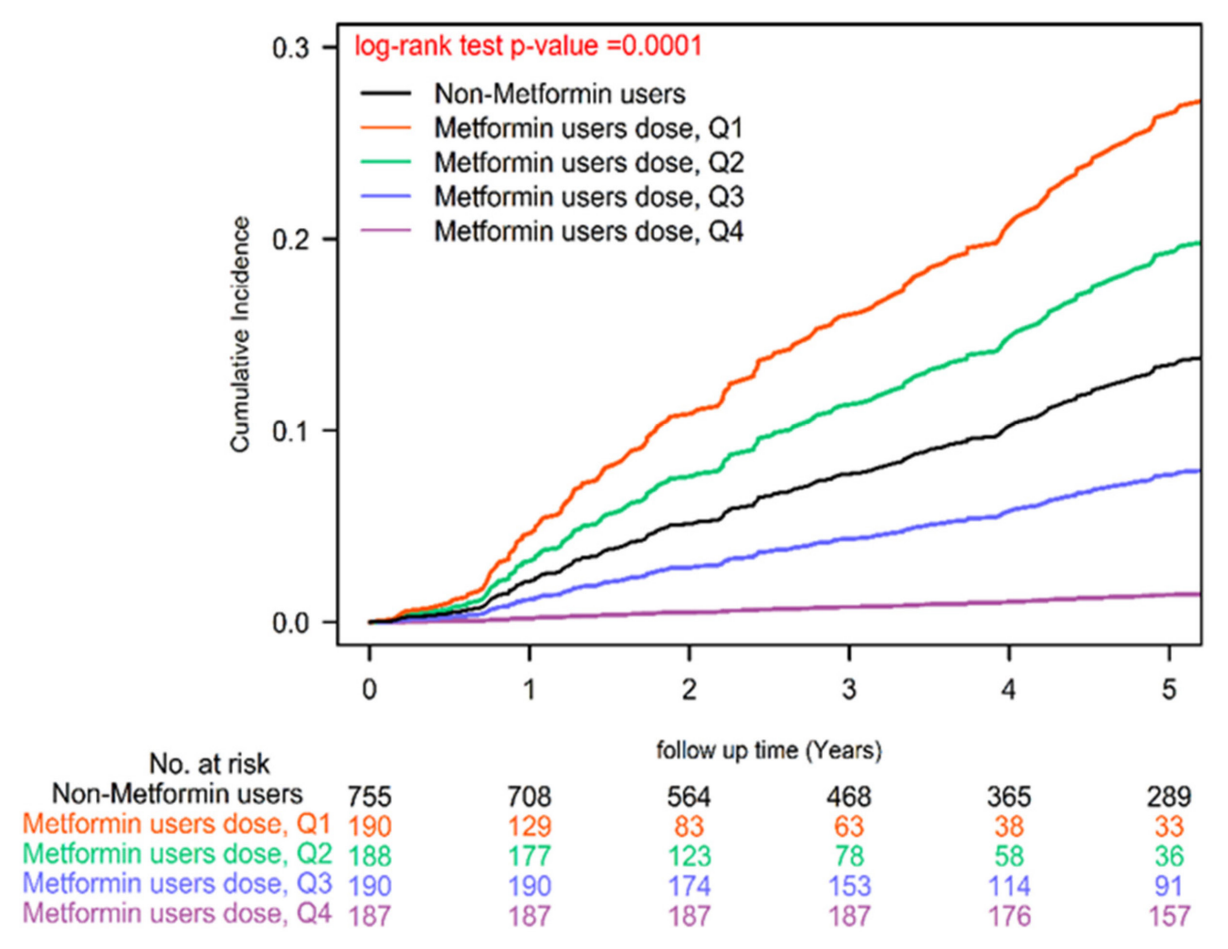
Cumulative Incidences of Herpes Zoster Infection in Patients with Type II Diabetes and Colon Cancer Undergoing Curative Standard Treatments: Varied Metformin Dosages.

To comprehensively account for potential confounding variables, our Cox regression models underwent meticulous adjustments, encompassing a wide array of covariates, including age, sex, income levels, urbanization degree, years since cancer diagnosis, AJCC clinical stage according to the 8th edition, numbers of antidiabetic drugs used, utilization of antidiabetic medications, aDCSI scores, presence of concurrent comorbidities, CCI scores, and other medications 39. Furthermore, we employed a time-dependent Cox hazards model to compare the incidence of herpes zoster infection between metformin users and nonusers, with rigorous adjustments for the aforementioned covariates. Recognizing the dynamic nature of metformin prescriptions, we diligently collected metformin usage data every 3 months, enabling precise characterization of metformin status as a time-dependent variable. To address potential biases, we categorized event-free person-years during follow-up periods devoid of metformin use for a minimum of 3 months as unexposed follow-up intervals. Poisson Regression analysis was employed to estimate the IRR of herpes zoster infection, and competing risk analysis was conducted to account for mortality risk. Cumulative incidences of herpes zoster infection were meticulously evaluated using the Kaplan-Meier method, and distinctions between metformin users and nonusers were scrutinized using a stratified log-rank test (Figure 1). Similarly, we estimated the incidence of herpes zoster infection according to various levels of cDDD using the Kaplan-Meier method, with distinctions assessed via a stratified log-rank test (Figure 2). All statistical analyses were executed utilizing SAS software (version 9.4; SAS Institute, Cary, NC, USA), ensuring the robustness and rigor of our data analysis.

## Results

Following PSM, our study encompassed a cohort of 1,510 individuals diagnosed with both T2DM and colon adenocarcinoma, devoid of distant metastases, who underwent standard treatments spanning from 2008 to 2018. The average age at the time of T2DM diagnosis mirrored at 65.88 years for both metformin users and nonusers. Notably, a meticulous examination revealed an impeccably balanced distribution of all covariates between the two groups. This equilibrium underscored the triumph of our adjustment procedure in attaining covariate parity, as distinctly evidenced in Table 1, with every absolute standardized mean difference comfortably resting below 0.1, reinforcing the robustness of our analysis 40.

### Metformin use and dose-dependent protective effects for herpes zoster infection

Our analysis unveiled a significant reduction in herpes zoster infection risk among colon cancer patients undergoing standard treatments who were also metformin users, as reflected by a noteworthy adjusted hazard ratio (aHR) of 0.69 (95% CI: 0.51 to 0.93; Table 2). This finding was consistently supported by a statistically significant log-rank test result (P=0.0161; Figure 1). Moreover, our Cox regression analysis illuminated a dose-dependent relationship between metformin use and diminishing herpes zoster infection risk. The examination of cDDD of metformin revealed a systematic dose-response pattern, with progressively declining aHRs observed across quartiles (0.10, 0.55, 0.92, and 0.94 for quartiles 4, 3, 2, and 1, respectively) when compared to individuals who had never utilized metformin (P for trend < 0.0001). These compelling findings, visually represented in Figure 2 (P = 0.0001), offer robust evidence for the dose-dependent protective effects of metformin against herpes zoster infection.

### Competing risk of mortality

Upon meticulous adjustment for all covariates, as delineated in Table 1, using a dynamic Cox proportional regression model that accounts for the competing risk of mortality (Table 3), we determined that the aHR for herpes zoster infection within our metformin user cohort stood at 0.70 (95% CI: 0.51 to 0.65). Notably, a substantial and dose-dependent reduction in herpes zoster infection risk was evident across various quartiles of cDDD of metformin (P for trend < 0.0001). The aHRs for quartiles 4, 3, 2, and 1 of cDDD were as follows: 0.12 (95% CI: 0.05 to 0.25), 0.58 (95% CI: 0.35 to 0.95), 0.96 (95% CI: 0.87 to 1.13), and 0.97 (95% CI: 0.91 to 1.33), respectively, when contrasted with individuals who had never initiated metformin therapy.

### IR and IRRs of herpes zoster infection

Within our cohort of individuals with colon cancer and T2DM, we noted that 9.54% of those using metformin (72 individuals) and 13.77% of those not using metformin (104 individuals) experienced herpes zoster infections, marking a statistically significant difference (P=0.0449; Table 1). Metformin users exhibited lower IRs of herpes zoster infection compared to nonusers, as detailed in Table 4. An analysis of the IRRs indicated that metformin users had an IRR (95% CI) of 0.75 (0.56 to 0.97) relative to nonusers.

Specifically, the IR for herpes zoster infection among metformin users amounted to 210.17 per 10,000 person-years, whereas non-metformin users displayed an IR of 287.53 per 10,000 person-years. When stratified by quartiles of cDDD of metformin, the IRRs (95% CI) for herpes zoster infection in metformin users, compared with nonusers, were as follows: 0.13 (0.06 to 0.30) for quartile 4, 0.64 (0.39 to 0.94) for quartile 3, 1.11 (0.87 to 2.27) for quartile 2, and 1.12 (0.84 to 2.03) for quartile 1.

## Discussion

In the realm of devising immunosuppressive strategies for adults, especially those at heightened susceptibility to herpes zoster like solid organ transplant recipients and certain cancer or autoimmune disorder patients, the role of zoster vaccination warrants thoughtful consideration 41-43. It's worth noting the financial implications associated with these vaccines 44, 45, particularly as they lack coverage under Taiwan's National Health Insurance program. The primary obstacles reported by physicians and patients in relation to vaccination were predominantly financial in nature 44, 45. Moreover, the imperative for zoster vaccination may not be universally applicable across distinct cancer types, given varying levels of zoster infection risk 46, 47. Thus, our investigation delivers valuable insights by not only presenting the incidence rate of zoster infection in colon cancer patients undergoing standard treatments but also by calculating the incidence rate ratio for zoster infection (Table 4). Furthermore, we affirm the protective impact of the cost-effective and well-tolerated medication, metformin, in curtailing the risk of zoster infection (Table 2-3). These findings significantly enrich our comprehension of herpes zoster risk management in cancer patients, potentially alleviating concerns regarding vaccination costs and broadening therapeutic horizons. Despite the presence of vaccines and pharmaceutical interventions for herpes zoster, this infection remains a substantial healthcare challenge on a global scale 15. Notably, our research uncovers a substantial association between metformin usage and a diminished risk of herpes zoster infection. Furthermore, an elevated cumulative metformin dose correlates with a progressively reduced zoster risk (Table 2). These observations suggest that metformin might offer a promising avenue for alleviating the burden of zoster, especially among colon cancer patients with T2DM who are undergoing standard treatments. Of note, our study stands as the inaugural demonstration of a significant reduction in the risk of herpes zoster infection among metformin users in this specific cohort, yielding an aHR of 0.69. This protective effect of metformin exhibits a dose-dependent trend, with consistently lower aHRs across quartiles of cDDD of metformin. Even subsequent to meticulous adjustments for competing mortality risks, the aHR for herpes zoster infection among metformin users maintains its significant reduction at 0.70, underscoring the potential benefits of metformin in mitigating the risk of herpes zoster in colon cancer patients with T2DM. Additionally, metformin users display a lower incidence rate of herpes zoster infection (9.54%) compared to nonusers (13.77%), with an IRR of 0.75, further accentuating the protective role of metformin. Stratified analysis by cDDD quartiles consistently unveils a dose-response relationship.

Metformin holds promise as a means to alleviate the burden of herpes zoster, particularly in colon cancer patients with T2DM undergoing standard treatments (Table 2-4 and Figure 1). This potential is rooted in a multifaceted array of mechanisms. Firstly, Metformin exerts immunomodulatory effects by activating AMP-activated protein kinase, bolstering T-cell immunity 22-24. This enhanced immune response can lower the risk of Zoster reactivation and mitigate infection severity 22-24. Secondly, Metformin's anti-inflammatory properties come into play by reducing the secretion of proinflammatory cytokines, thus mitigating the typical inflammatory response triggered by viral infections like Zoster 48-50. Thirdly, one potential mechanism by which Metformin may reduce the risk of herpes zoster in colon cancer patients with T2DM undergoing standard treatments is its impact on immune modulation 51, 52. The tumor itself and cancer treatments, such as chemotherapy and radiation therapy, can induce immune suppression 7, 8, rendering patients more vulnerable to infections, including the reactivation of the VZV responsible for Zoster 11. Additionally, metformin's ability to effectively control blood glucose levels indirectly enhances immune function, as hyperglycemia compromises immune responses 53. Crucially, the observed dose-dependent effect of metformin on reducing Zoster infection risk implies that a cumulative exposure threshold is necessary for its significant protective impact. Lower doses may not induce substantial immunomodulation, as seen in Q1 and Q2, whereas reaching a certain threshold (Q3 and Q4) is vital to unlock metformin's full potential in mitigating Zoster risk. In essence, metformin's multifaceted influence on immunity, inflammation, and glycemic control collectively positions it as a valuable candidate for reducing the risk of Zoster infection in colon cancer patients with T2DM undergoing standard treatments.

In addition to its protective effects against herpes zoster, metformin offers several direct and indirect clinical benefits for colon cancer patients. Metformin has demonstrated antineoplastic properties, potentially inhibiting cancer cell proliferation and inducing apoptosis in colorectal cancer cells 54, which can enhance overall treatment outcomes. Furthermore, metformin's ability to improve metabolic control is particularly beneficial for colon cancer patients with T2DM, as optimal glycemic control supports overall health and may reduce cancer-related complications 55. Metformin's anti-inflammatory properties, through the reduction of proinflammatory cytokines, can mitigate inflammation associated with both cancer and its treatment, thereby improving patient well-being 56. Additionally, metformin's role in immune modulation by activating AMP-activated protein kinase enhances T-cell immunity, which is crucial for patients undergoing immunosuppressive treatments like chemotherapy and radiation therapy 51. This multifaceted influence of metformin on immunity, inflammation, and glycemic control collectively positions it as a valuable therapeutic agent, not only for reducing the risk of herpes zoster infection but also for potentially lowering the risk of cancer recurrence and improving long-term survival rates in colon cancer patients 51, 54-56. These benefits underscore the importance of considering metformin as part of a comprehensive management strategy for colon cancer patients with T2DM.

In our study, we harnessed the manifold advantages of PSM, dose-dependent analysis, and time-dependent Cox regression, each serving a unique purpose in our complex real-world investigation. PSM, an invaluable tool in the absence of randomized controlled trials (RCTs) due to ethical considerations 57, 58, played a pivotal role in achieving covariate balance between metformin users and nonusers. Given the impracticality of mandating medication use or vaccination in real-world settings, PSM emulated the random assignment scenario of RCTs within the confines of observational data, bolstering the internal validity of our findings 58. Notably, Table 1 showcases the meticulous equilibrium achieved in all confounding factors between the case and control groups post PSM, underscoring the robustness of our analytical framework. Dose-dependent analysis unveiled a dose-response relationship between metformin dosage and herpes zoster risk. This nuanced exploration provided critical insights into the optimal metformin dosage required for risk reduction, shedding light on the potential for tailored treatment regimens. Time-dependent Cox regression emerged as a dynamic analytical approach 59, accounting for variations in metformin exposure over the study duration. As metformin use fluctuated throughout the observation period, this method provided a real-time assessment of its influence on herpes zoster risk 59. It enabled us to categorize individuals based on their evolving metformin exposure status, ensuring a precise representation of the medication's effects over time. To fortify the robustness of our findings, we incorporated competing risk analysis, which factored in the risk of mortality 60-62. This was especially crucial in a cohort of patients grappling with underlying conditions such as cancer and diabetes, where mortality is a significant consideration. By considering these competing risks, we bolstered the credibility and reliability of our results (Table 3). Taken together, our utilization of PSM, dose-dependent analysis, time-dependent Cox regression, and competing risk analysis collectively fortified the rigor and comprehensiveness of our study. These methodological choices allowed us to derive meaningful conclusions regarding the intricate relationship between metformin use and the risk of herpes zoster infection in colon cancer patients with T2DM undergoing standard treatments, offering valuable insights to the medical community.

Our study reveals that metformin significantly reduces the risk of herpes zoster infection in colon cancer patients with T2DM undergoing standard treatments, with a dose-dependent protective effect. This finding has several clinical implications and applications. Firstly, metformin's ability to lower herpes zoster incidence can improve patient outcomes by preventing complications like postherpetic neuralgia, thus enhancing the quality of life for these patients. Secondly, as a cost-effective and well-tolerated medication, metformin offers a financially viable alternative to vaccination, particularly in resource-limited settings. Integrating metformin into clinical guidelines for managing colon cancer patients with T2DM could optimize both glycemic control and herpes zoster prevention, necessitating collaboration among oncologists, endocrinologists, and primary care providers. The dose-dependent nature of its protective effect also supports a personalized medicine approach, allowing clinicians to tailor therapy based on individual patient risk profiles and tolerance levels. Moreover, this study suggests broader preventive applications of metformin in immunocompromised patients due to its immunomodulatory and anti-inflammatory properties. In summary, our research underscores the potential of metformin as a multifaceted therapeutic agent, enhancing patient outcomes, providing a cost-effective preventive strategy, and supporting personalized medicine, thereby contributing significantly to the holistic management of colon cancer patients with T2DM.Our study emphasizes the potential of metformin in reducing the risk of herpes zoster infection in colon cancer patients with Type 2 Diabetes Mellitus (T2DM) undergoing standard treatments. The protective effect is dose-dependent, suggesting that metformin could be a valuable addition to herpes zoster risk management strategies in this patient population.

Our study boasts several key strengths that enhance its credibility and contribution to the field. It leverages Taiwan's NHIRD and TCRD, providing a robust dataset for a population-based cohort study 29-31, 63. The integration of these databases with Taiwan's Death Registry enables precise determination of cancer treatments, stages, vital status, and causes of death 29-31, 63. Notably, our study exclusively focuses on a well-defined cohort of colon adenocarcinoma patients without distant metastasis who received standard treatments, closely aligning with established NCCN guidelines, thereby increasing its specificity and relevance. A significant strength lies in the rigorous application of PSM to balance covariates between metformin users and nonusers. In the absence of RCTs due to ethical constraints, PSM serves as a powerful tool to emulate random assignment within observational data 57, 58, significantly enhancing the internal validity of our findings. This success is evident in the well-balanced distribution of all confounding factors between the two groups (Table 1). Furthermore, our study offers novel insights into the relationship between metformin use and the risk of herpes zoster infection in colon cancer patients with T2DM undergoing standard treatments. It is the first to demonstrate a significant reduction in the risk of herpes zoster infection among metformin users within this specific cohort, revealing a dose-dependent effect that persists even after adjusting for competing mortality risks. These findings present a compelling case for metformin as a potential avenue to alleviate the burden of herpes zoster in this vulnerable population, significantly contributing to our understanding of herpes zoster risk management, particularly in the context of cancer and diabetes care. The innovative approach employed to analyze metformin's protective effects in a real-world, complex clinical scenario underscores the novelty and significance of this study.

Our study, while valuable, comes with several limitations inherent to its retrospective nature and reliance on administrative health data, which may contain errors or missing information. Although we diligently employed PSM to mitigate selection bias, the influence of unmeasured variables, such as lifestyle factors, on both metformin use and herpes zoster risk cannot be entirely ruled out. Despite comprehensive covariate adjustment, concerns regarding the presence of unmeasured confounders persist. The generalizability of our findings is constrained as our study exclusively focuses on colon adenocarcinoma patients with T2DM in Taiwan. Moreover, metformin use was defined solely based on pharmacy claims, without accounting for actual adherence. The termination of the follow-up period in December 2021 raises the potential for missing long-term effects. However, it's worth noting that several cancers were associated with an increased risk of zoster, particularly within the first 2 years after diagnosis 64. Therefore, the mean follow-up time of 5.44 years for the cohort is deemed sufficient for estimating the protective effects of metformin against herpes zoster in cancer patients. Lastly, our study did not delve into the specific mechanisms underpinning metformin's protective effect against herpes zoster. Despite these limitations, our research significantly contributes by highlighting an association between metformin use and reduced herpes zoster risk within this specific cohort. This association warrants further exploration and confirmation in more extensive and diverse populations and settings, with an emphasis on investigating the underlying mechanisms.

Our population-based cohort study reveals metformin's potential to reduce herpes zoster risk in colon cancer patients with T2DMundergoing standard treatments, with a significant dose-dependent effect as cumulative defined daily doses increase. This suggests metformin, a well-established diabetes medication, could offer an innovative approach to easing herpes zoster's burden in this vulnerable group. While further research is needed to understand the precise mechanisms, our findings underscore metformin's clinical significance in enhancing the well-being of these patients, informing herpes zoster risk management, and highlighting the broader potential of repurposing existing medications to address complex healthcare challenges.

## Figures and Tables

**Table 1:** Propensity Score-Matched Characteristics of Metformin Users and Non-Users Among Patients with Type II Diabetes and Colon Cancer Undergoing Curative Standard Treatments

	Non-Metformin		Metformin		ASMD
	N=755		N=755		
	N	%	N	%	
Age (mean ± SD)	65.88 ± 9.63		65.88 ± 9.63		
Age, median (IQR), y	69.00 (61.00,76.00)		69.00 (61.00,76.00)		
Age group, years					0.0000
<60	189	25.03%	189	25.03%	
60-65	146	19.34%	146	19.34%	
66-70	134	17.75%	134	17.75%	
≥71	286	37.88%	286	37.88%	
Sex					0.0242
Female	320	42.38%	329	43.58%	
Male	435	57.62%	426	56.42%	
Income level (NTD)					0.0000
Low income	10	1.32%	10	1.32%	
≤20 000	652	86.36%	652	86.36%	
20 001-30 000	51	6.75%	51	6.75%	
30 001-45 000	33	4.37%	33	4.37%	
>45 000	9	1.19%	9	1.19%	
Urbanization					0.0084
Rural	231	30.60%	234	30.99%	
Urban	524	69.40%	521	69.01%	
Years of Cancer Diagnosis					0.0000
2008-2011	130	17.22%	130	17.22%	
2012-2016	328	43.44%	328	43.44%	
2017-2020	297	39.34%	297	39.34%	
AJCC clinical Stage, 8^th^ edition					0.0590
I	237	31.39%	233	30.86%	
II	157	20.79%	158	20.93%	
IIIA	16	2.12%	23	3.05%	
IIIB	106	14.04%	106	14.04%	
IIIC	239	31.66%	235	31.13%	
Types of antidiabetic drugs used (Diabetes severity)					0.0041
0	314	41.59%	312	41.32%	
1	185	24.50%	187	24.77%	
2	160	21.19%	162	21.46%	
3	69	9.12%	70	9.27%	
≥4	27	3.58%	24	3.18%	
Antidiabetic drug					
Insulin	94	12.45%	97	12.86%	0.0013
Sulfonylureas	315	41.72%	311	41.19%	0.0011
SGLT2 inhibitors	12	1.59%	11	1.46%	0.0009
Alpha-glucosidase inhibitors	47	6.23%	46	6.09%	0.0012
Thiazolidinediones	25	3.31%	28	3.71%	0.0007
GLP-1 agonists	9	1.19%	8	1.06%	0.0016
DPP4 inhibitors	43	5.70%	47	6.23%	0.0021
Diabetes severity					
aDCSI Score (mean ± SD)	1.14 ± 1.56		1.14 ± 1.50		
Median (IQR, Q1-Q3)	1.00 (0.00,2.00)		1.00 (0.00,2.00)		
aDCSI Score					
0	327	43.31%	326	43.18%	0.0008
1	177	23.44%	178	23.58%	0.0003
2	126	16.69%	127	16.82%	0.0009
3	69	9.14%	70	9.27%	0.0002
≥4	56	7.42%	54	7.15%	0.0004
CCI Scores					
Mean (SD)	3.46 ± 3.00		3.41 ± 3.20		
Median (Q1-Q3)	2.00 (0.00,6.00)		2.00 (1.00,6.00)		
CCI Scores					0.0780
0	191	25.30%	166	21.99%	
≥1	564	74.70%	589	78.01%	
Coexisting comorbidities					
Hypertension	571	75.63%	572	75.76%	0.0030
Hyperlipidemia	395	52.32%	400	52.98%	0.0132
Chronic Obstructive Pulmonary Disease	205	27.15%	198	26.23%	0.0208
Alcohol-related disorders	38	5.03%	29	3.84%	0.0578
Coronary artery disease	273	36.16%	271	35.89%	0.0056
Stroke	201	26.62%	194	25.70%	0.0209
Heart failure	75	9.93%	70	9.27%	0.0224
Peripheral vascular disease	76	10.07%	71	9.40%	0.0226
Chronic kidney disease	30	3.97%	23	3.05%	0.0500
Depression	139	18.41%	133	17.62%	0.0206
Anxiety	66	8.74%	68	9.01%	0.0095
Dementia	26	3.44%	29	3.84%	0.0214
Psychosis	59	7.81%	45	5.96%	0.0731
Rheumatoid arthritis	25	3.31%	23	3.05%	0.0148
Liver Cirrhosis	257	34.04%	253	33.51%	0.0112
Systemic Lupus Erythematosus	13	1.72%	17	2.25%	0.0380
Obesity	240	31.79%	244	32.32%	0.0086
Medication Use					
Statin	287	38.01%	294	38.94%	0.0191
Aspirin	375	49.67%	391	51.79%	0.0424
Immunosuppressant	5	0.66%	6	0.79%	0.0153
Corticosteroids for systemic use	626	8.91%	634	83.97%	0.0285
Metformin (cDDD)					
Never used	755	100.00%	0	0.00%	
Q1(153)	0	0.00%	190	25.17%	
Q2 (411)	0	0.00%	188	24.90%	
Q3 (962)	0	0.00%	190	25.17%	
Q4 (> 962)	0	0.00%	187	24.77%	
DDD					
<1	755	100.00%	723	95.76%	
≥1	0	0.00%	32	4.24%	
Primary Outcome					*P* value
Herpes zoster infection	104	13.77%	72	9.54%	0.0449

**Abbreviations:** aDCSI, adapted Diabetes Complications Severity Index; DDD, defined daily dose; cDDD, cumulative defined daily dose; DM, diabetes mellitus; T2DM, Type 2 diabetes mellitus; Q, quartile; CCI, Charlson comorbidity index; ASMD, absolute standardized mean difference; N, Numbers; NTD, New Taiwan Dollars; SD, Standard deviation; IQR, interquartile range.

**Table 2:** Hazard Ratios for Herpes Zoster Infection in Patients with Type II Diabetes and Colon Cancer Undergoing Curative Standard Treatments, Stratified by Varied Metformin Dosages

	Crude HR (95%CI)		P-value	Adjusted HR (95%CI)		P-value
Metformin (ref. Never-users)						
Users	0.75	(0.56, 1.01)	0.0557	0.69	(0.51, 0.93)	0.0161
Metformin DDD (ref. no)						
Q1	1.25	(0.83, 3.24)	0.1762	0.94	(0.84, 3.14)	0.1023
Q2	1.36	(0.82, 2.40)	0.2407	0.92	(0.87, 1.35)	0.0665
Q3	0.63	(0.39, 1.03)	0.0661	0.55	(0.33, 0.9)	0.0185
Q4	0.12	(0.05, 0.28)	<0.0001	0.10	(0.04, 0.22)	<0.0001
*P for trend*						<0.0001

**Abbreviations:** Q, quartile; HR, hazard ratio; CI, confidence interval. ^*^Adjusted for all covariates shown in Table 1 using a Cox proportional regression model.

**Table 3:** Competing Risk Analysis of Hazard Ratios for Herpes Zoster Infection Among Patients with Type II Diabetes and Colon Cancer Undergoing Curative Standard Treatments, Stratified by Varied Metformin Dosages

		
	Crude HR (95%CI )		P-value	Adjusted HR (95%CI )		P-value
Metformin (ref. Never-users)						
Users	0.75	(0.56, 1.01)	0.0557	0.70	(0.51, 0.95)	0.0210
Metformin DDD (ref. no)						
Q1	1.25	(0.83, 3.24)	0.1762	0.97	(0.91, 1.33)	0.1527
Q2	1.36	(0.82, 2.40)	0.2407	0.96	(0.87, 1.13)	0.1753
Q3	0.63	(0.39, 1.03)	0.0661	0.58	(0.35, 0.95)	0.0310
Q4	0.12	(0.05, 0.28)	<0.0001	0.12	(0.05, 0.25)	<0.0001
*P for trend*						<0.0001

**Abbreviations:** Q, quartile; HR, hazard ratio; CI, confidence interval. ^*^Adjusted for all covariates shown in Table 1 using a Cox proportional regression model with competing risk of mortality.

**Table 4:** Incidence Rate and Incidence Rate Ratio of Herpes Zoster Infection in Patients with Type II Diabetes and Colon Cancer Undergoing Curative Standard Treatments

Variables	Events	Person-years	IR (10,000 person-years)	IRR	95% CI for IRR	*P*
Metformin						
Never-users	104	3,617	287.53	Reference		
Users	72	3,793	210.17	0.75	(0.56, 0.97)	0.0434
Metformin						
Never-users	104	3,617.0	287.53	Reference		
Q1	27	517.1	580.14	1.12	(0.84, 2.03)	0.1807
Q2	24	632.8	426.68	1.11	(0.87, 2.27)	0.1676
Q3	16	1,039.6	182.76	0.64	(0.39, 0.94)	0.0423
Q4	5	1,603.7	37.41	0.13	(0.06, 0.30)	<0.0001

**Abbreviations:** Q, quartile; IR, incidence rate; IRR, incidence rate ratio; and CI, confidence interval.
